# Supplementary material for: Synergy of ferroelectric polarization and oxygen vacancy to promote CO2 photoreduction
Source: Nat Commun. 2021 Jul 28;12:4594. doi: 10.1038/s41467-021-24882-3 (PMC8319429; doi:10.1038/s41467-021-24882-3)
Supplement: Supplementary file 1 — Supporting information [file 41467_2021_24882_MOESM1_ESM.pdf]

Supplementary information

Synergy of Ferroelectric Polarization and Oxygen Vacancy to Promote CO_2 Photoreduction

Hongjian Yu[1], Fang Chen[1], Xiaowei Li[1], Hongwei Huang[1], Qiuyu Zhang[1], Shaoqiang Su[3], Keyang Wang[2], Enyang Mao[1], Bastian Mei[3], Guido Mul[3], Tianyi Ma[4]* and Yihe Zhang[1]**

[1]Beijing Key Laboratory of Materials Utilization of Nonmetallic Minerals and Solid Wastes, National Laboratory of Mineral Materials, School of Materials Science and Technology, China University of Geosciences, Beijing 100083, China

[2]The department of mechanics and engineering science, college of civil engineering and mechanics, Lanzhou University, Lanzhou, Gansu 730000, P.R. China

[3]Photocatalytic Synthesis Group, MESA+ Institute for Nanotechnology, University of Twente, P.O. Box 217, 7500 AE Enschede, The Netherlands

[4]Centre for Translational Atomaterials, Swinburne University of Technology, Hawthorn, Victoria, 3122, Australia

[*] Corresponding authors: hhw@cugb.edu.cn, tianyima@swin.edu.au, zyh@cugb.edu.cn

1. Supplementary Figures

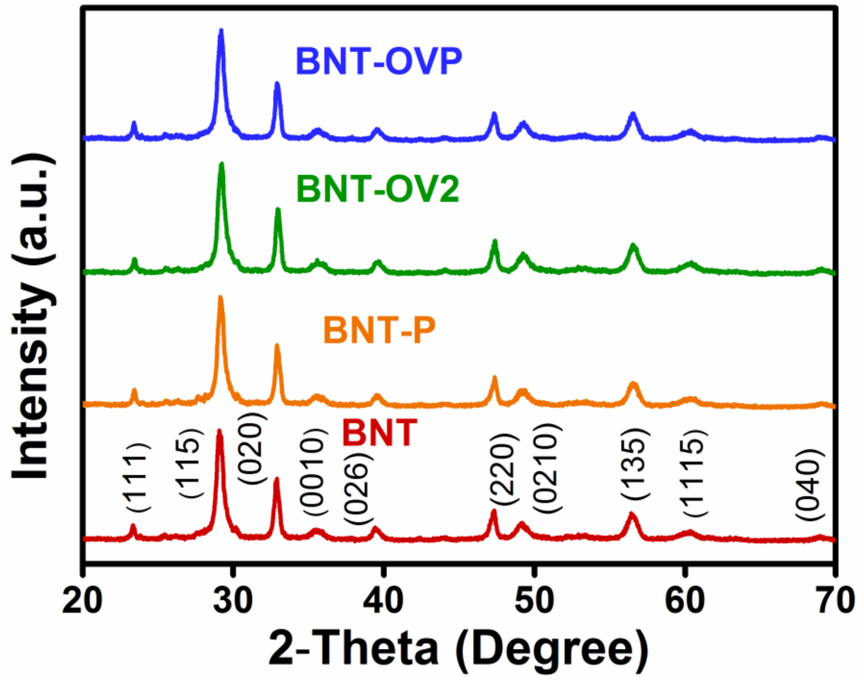

Supplementary Figure 1. XRD patterns of BNT, BNT-P, BNT-OV2 and BNT-OVP.

The diffraction peaks of BNT are well indexed into orthorhombic phase Bi_3TiNbO_9 (ICSD#16487) without any other peaks. Notably, BNT-P, BNT-OV2 and BNT-OVP are all pure phase without any change for diffraction peaks, indicating corona poling and oxygen vacancy creation do not change the crystal structure of Bi_3TiNbO_9 nanosheets.

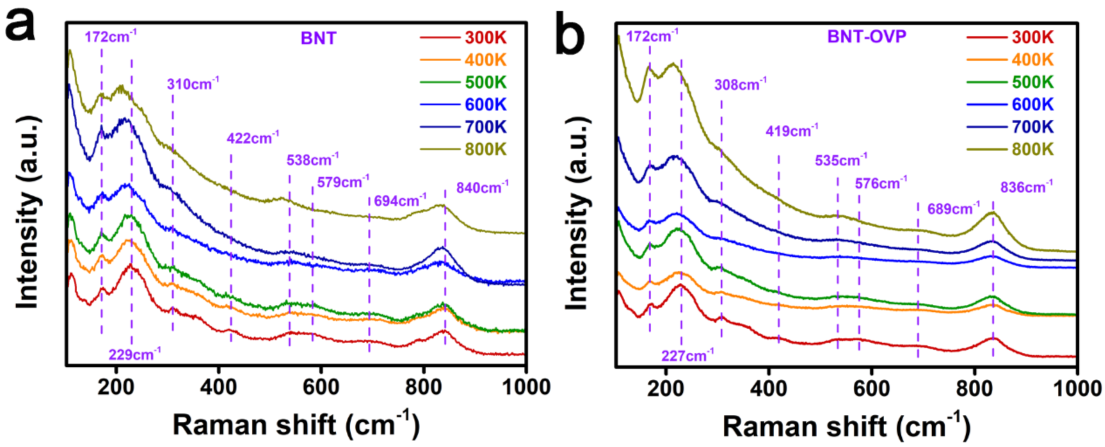

Supplementary Figure 2. (a, b) Raman spectra of BNT and BNT-OVP at different temperatures.

The Raman spectroscopic test at different temperatures has been conducted to distinguish the differences of metal-oxygen vibrations. In general, in layered Bi-based perovskite materials, the vibration modes below 200 cm^{-1} are assigned to the vibrations of the Bi^{3+} ions at Bi_2O_2 layers or as A-site in perovskite slabs, and the modes above 200 cm^{-1} are ascribed to the octahedral O-B-O and B-O vibrations (here A and B indicates the A and B sites in ABO_3 unit, respectively). [1,2] As shown in Supplementary Figure 2, there is no obvious shift for the modes below 200 cm^{-1}, and the modes above 200 cm^{-1} exhibit a slight shift to low-frequency position, indicating that oxygen vacancies occurred in the perovskite [$TiNbO_7$] slabs. As the temperature increases, some weak modes vanish especially in the wave number range of 200-900 cm^{-1}, which implies that its crystal structure and magnetic transition changes and the structural symmetry is improved. This phenomenon is attributed to the subtle change of crystalline phase from the ferroelectric $A2_1am$ to an intermediate symmetry as the number of Raman modes decrease upon heating. [1]

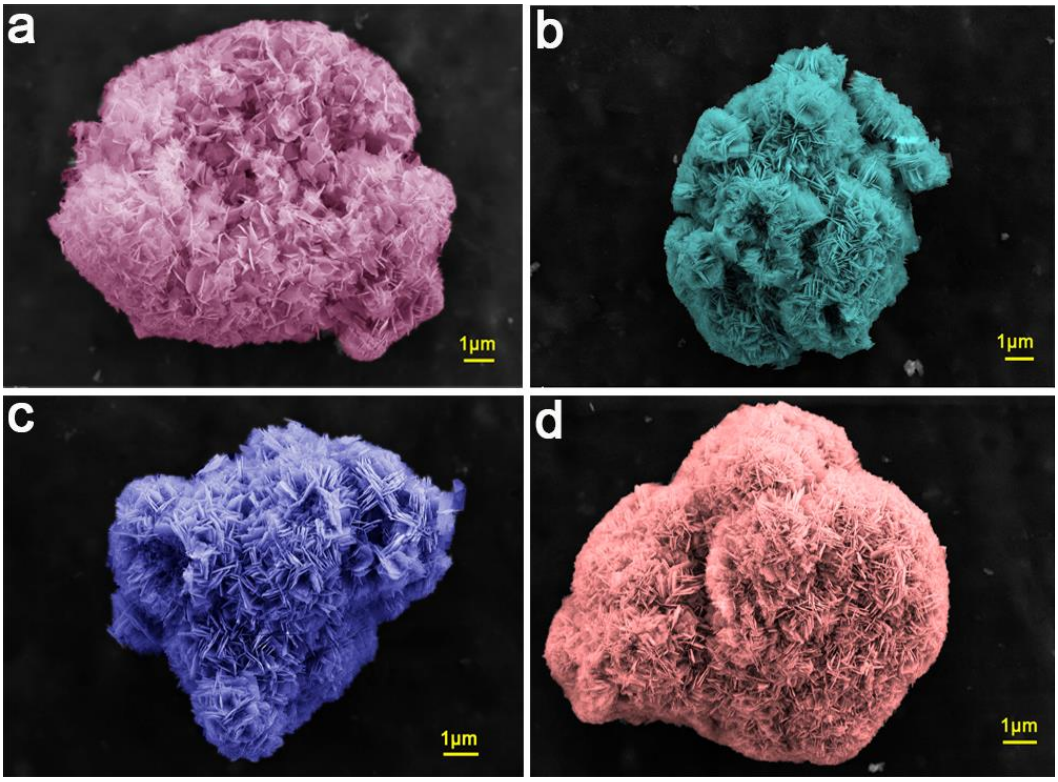

Supplementary Figure 3. SEM images of (a) BNT, (b) BNT-P, (c) BNT-OV2 and (d) BNT-OVP.

The morphologies of all the samples are analyzed by SEM. Namely, BNT diaplays the thickness of nanosheet to be 10-30nm. What's more, the microstructures of BNT-P, BNT-OV2

and BNT-OVP show no visible difference compared with BNT in good agreement with the above XRD result.

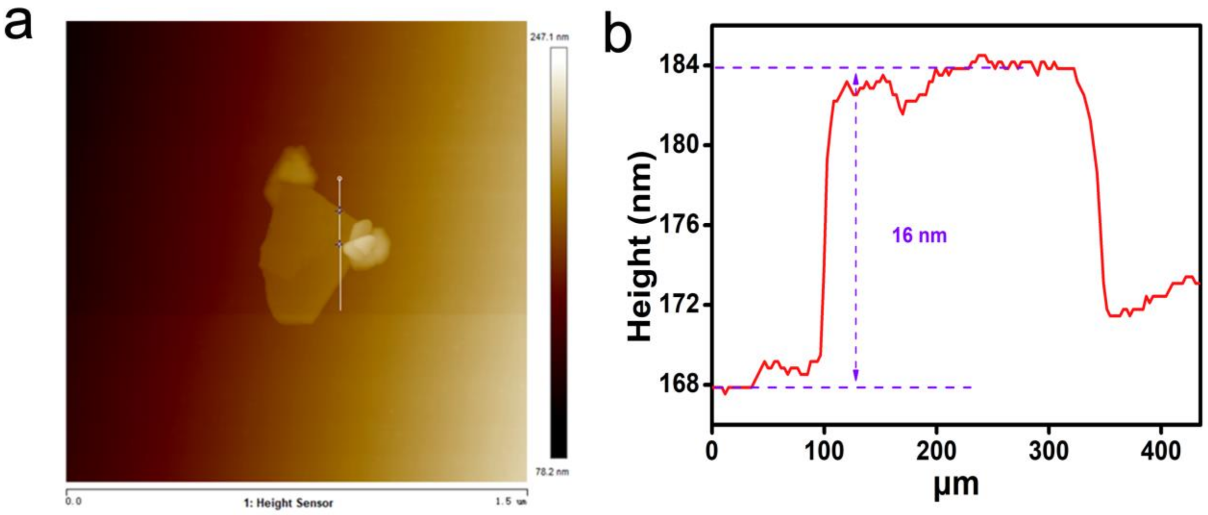

Supplementary Figure 4. (a) AFM image and (b) the corresponding height of BNT-OVP. Atomic force microscope (AFM) image further shows that thickness of the samples is 10-30 nm, which is consistent with the SEM results.

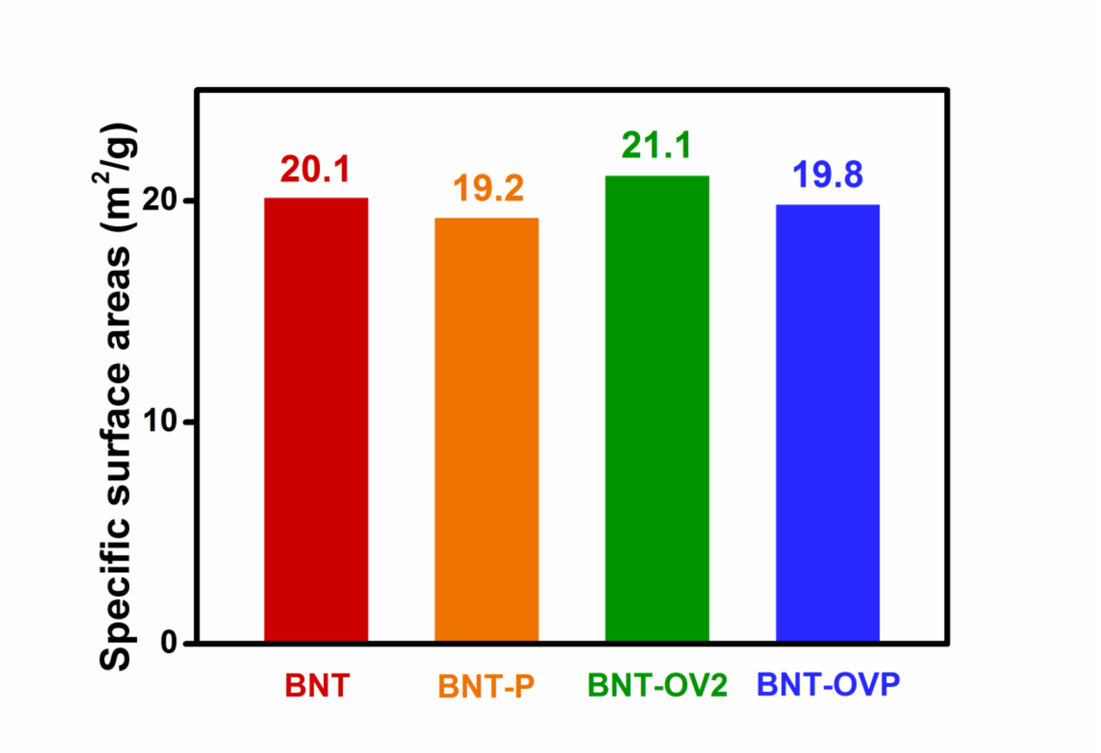

Supplementary Figure 5. BET specific surface area of BNT, BNT-P, BNT-OV2 and BNT-OVP.

The Brunauer, Emmett and Teller (BET) specific surface area of BNT, BNT-P, BNT-OV2 and BNT-OVP is 20.1, 19.2, 21.1 and 19.8 m^2/g, respectively. It can be concluded that the contribution of specific surface area to their photocatalytic activity difference can be ignored.

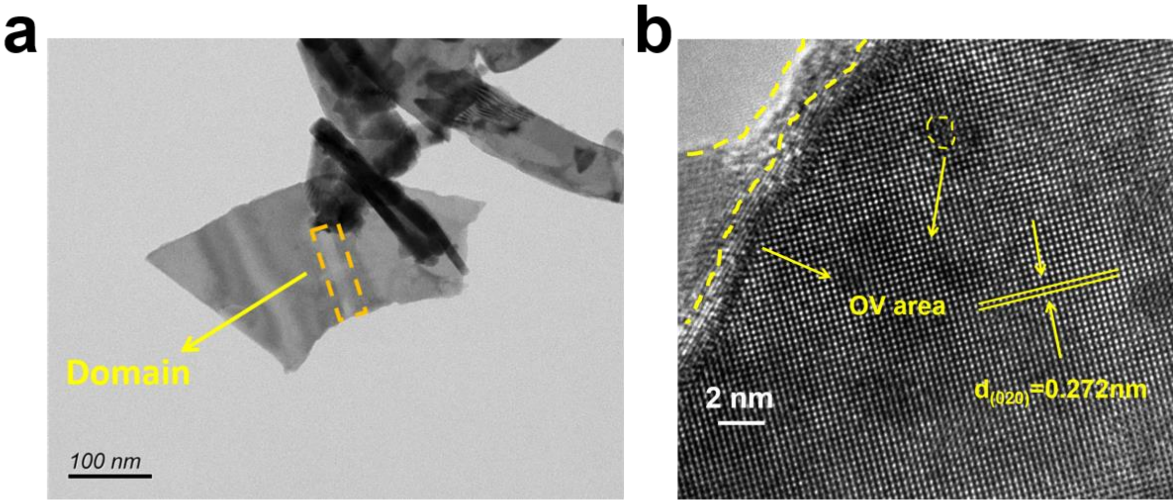

Supplementary Figure 6. (a)TEM image of BNT and (b) HRTEM images of BNT-OVP.

The transmission electron microscopy (TEM) image (Supplementary Figure 6a) displays the sample of BNT with a nanosheet structure, consistent with the SEM image. In addition, it is worth noticing from the HRTEM images (Supplementary Figure 6b) that BNT-OVP shows damaged edge and atomic deficiency demonstrating the existence of oxygen vacancies on the surface of BNT-OVP.

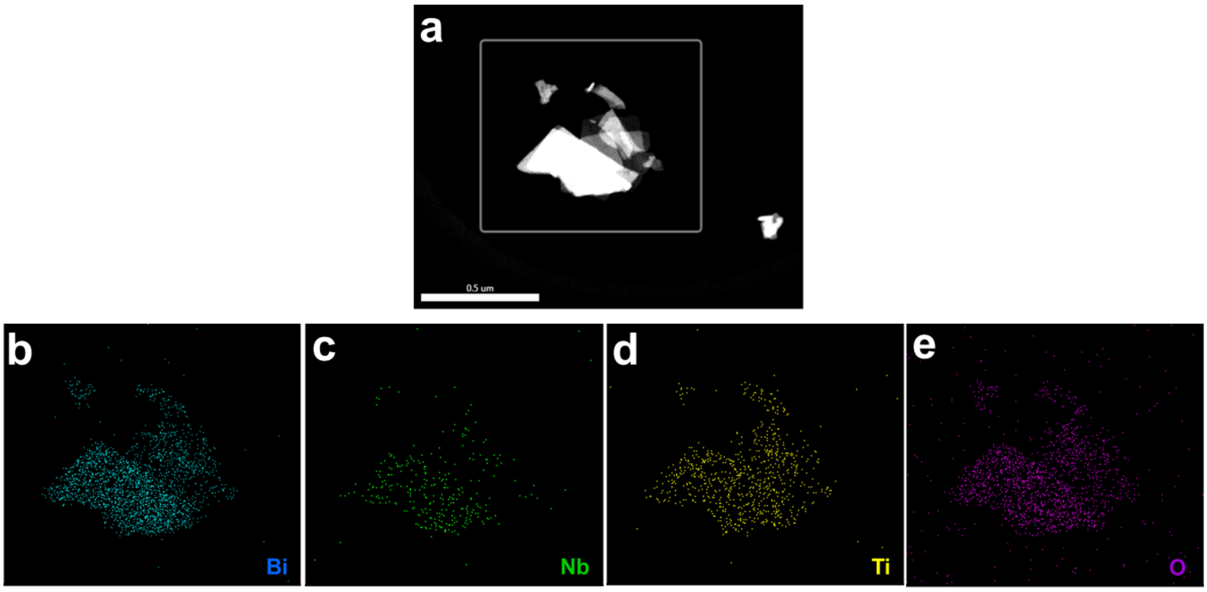

Supplementary Figure 7. (a) TEM image and the corresponding EDX elemental mappings

of Bi (b), Nb (c), Ti (d) and O (e) on the BNT-OVP.

TEM energy dispersive X-ray (EDX) elemental mapping indicates the homogeneous distribution of Bi, Nb, Ti and O across BNT-OVP nanosheets.

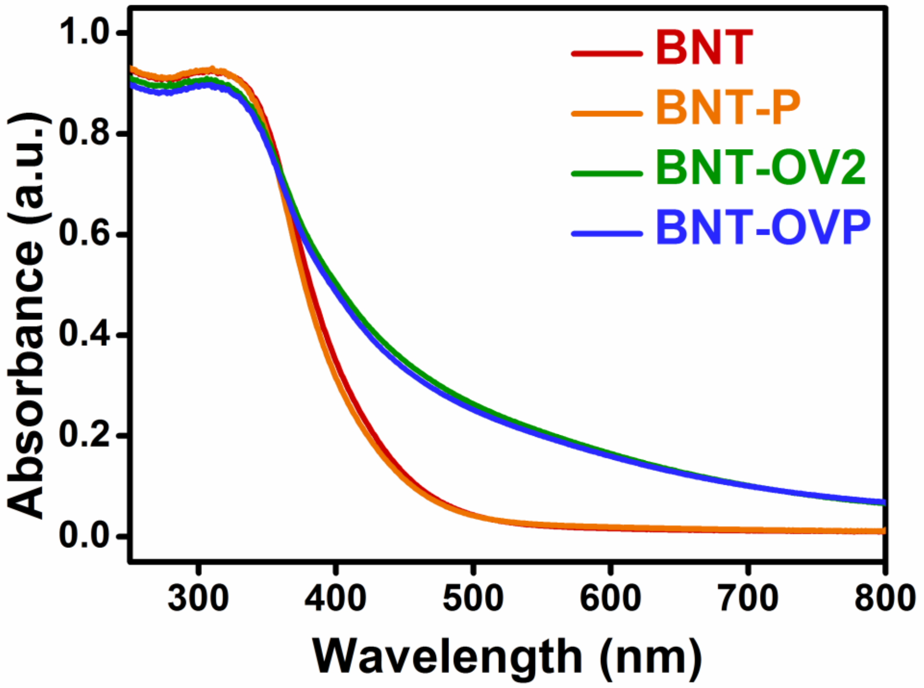

Supplementary Figure 8. UV/Vis diffuse reflectance spectra (DRS) of BNT, BNT-P, BNT-OV2 and BNT-OVP.

UV/vis diffuse reflectance spectra (DRS) demonstrates that BNT exhibit an absorption edge at around 400 nm which accords with the white color. Compared to BNT, the absorption edge of BNT-P displays no big difference, which illustrate that corona poling process has no effect on light absorption. Additionally, with the introduction of oxygen vacancies, the absorption range of BNT-OV2 and BNT-OVP was significantly extended after the introduction of OVs.

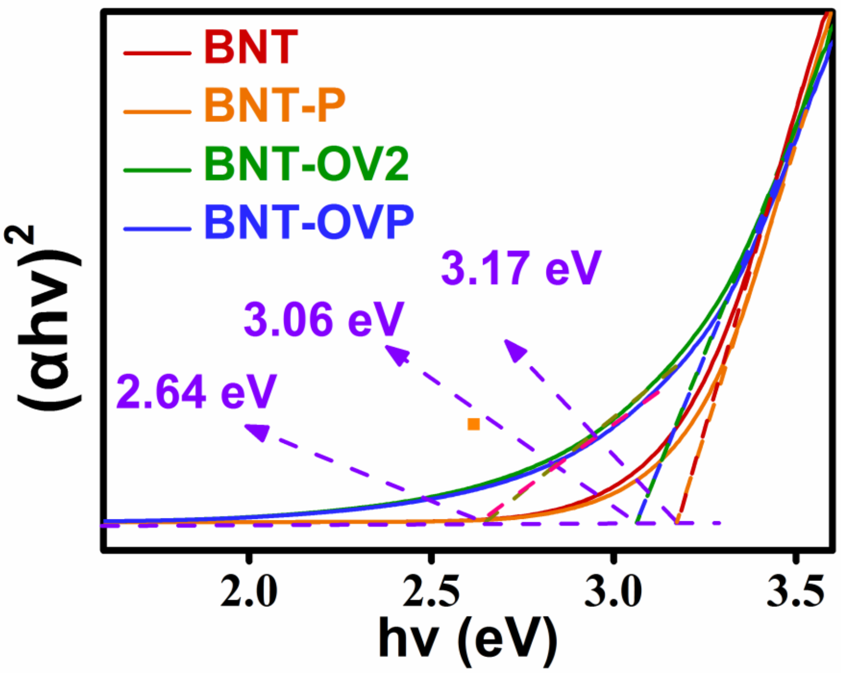

Supplementary Figure 9. Band gap of BNT, BNT-P, BNT-OV2 and BNT-OVP.

The bandgap energies of BNT and BNT-P are estimated from the plot of $(ah\upsilon)^{1/2}$ versus photon energy ($h\upsilon$) to be 3.17 eV. Notably, there are two band gaps for BNT-OV2 and BNT-OVP, which are ~3.06 and 2.64 eV. This is consistent with the fact that OVs always cause tail absorption.

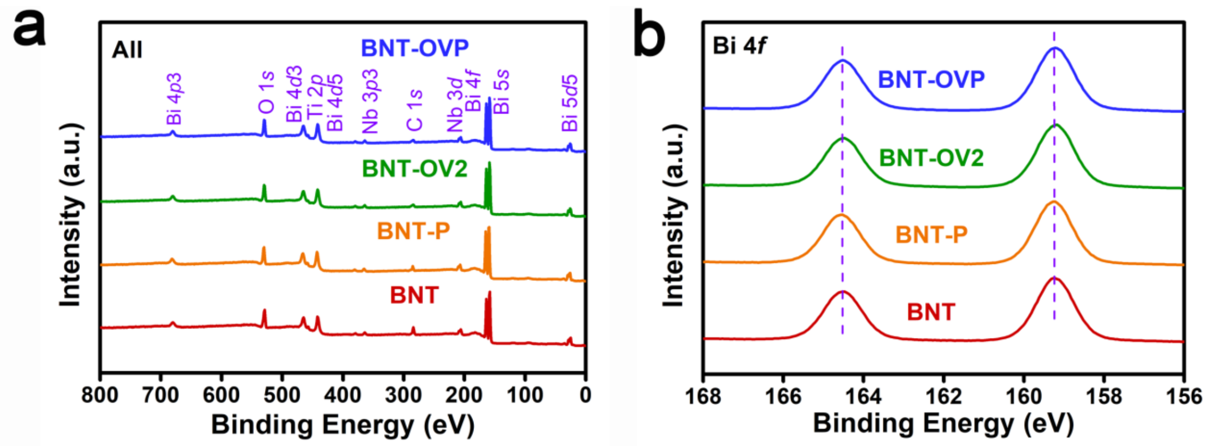

Supplementary Figure 10. (a) Survey XPS spectra, (b) Bi 4*f* XPS spectra of BNT, BNT-P, BNT-OV2 and BNT-OVP.

The surface composition and chemical states of related elements for all samples are analyzed

by X-ray photoelectron spectroscopy (XPS). Four constituent elements Bi 4*f*, Nb 3*d*, Ti 2*p* and O 1*s* all can be found in the survey XPS spectra for all samples (Supplementary Figure 10a). Meanwhile, the Bi 4*f* XPS spectra shows imperceptible shift (Supplementary Figure 10b), also suggests the less possibility of OVs appeared in [Bi_2O_2] layers.

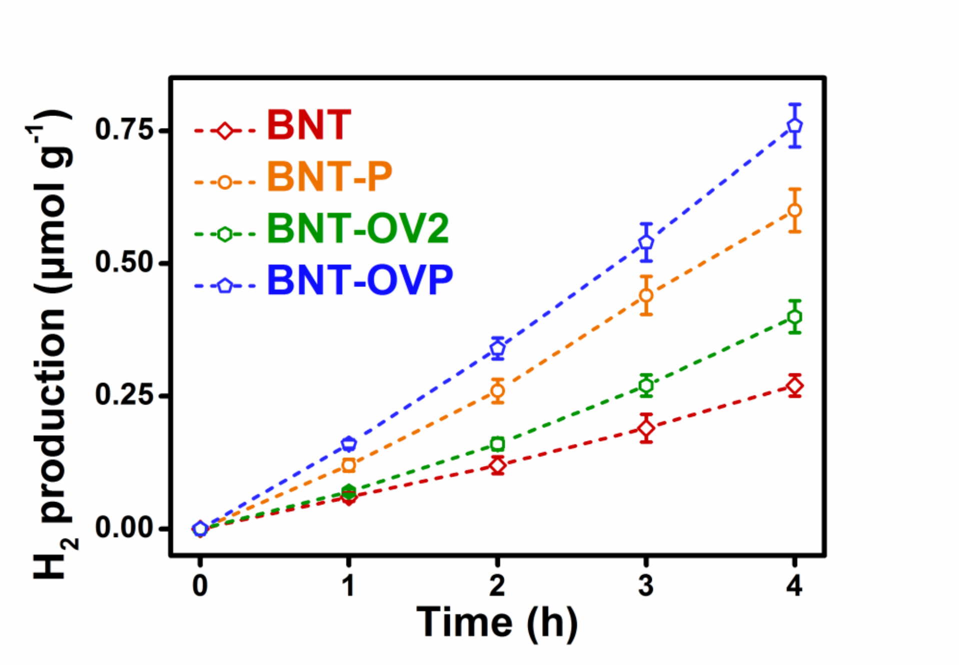

Supplementary Figure 11. H_2 production curves of BNT, BNT-P, BNT-OV2 and BNT-OVP under simulated solar light. Error bars represent the standard deviation.

H_2 production rates of BNT, BNT-P, BNT-OV2 and BNT-OVP are 0.07，0.15，0.11 and 0.19 μmol g^{-1} h^{-1}, respectively, under simulated solar light.

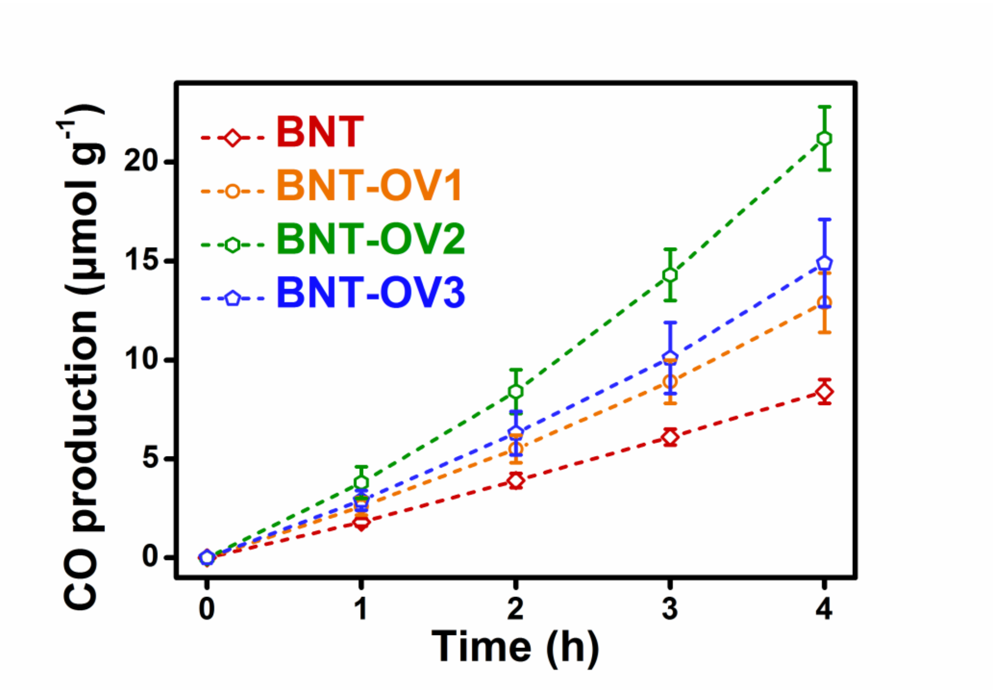

Supplementary Figure 12. CO production curves of different oxygen vacancy concentrations for BNT-OVX (X=1, 2, 3). Error bars represent the standard deviation.

Introduction of OVs on the surface of BNT can larely improve photocatalytic performance in CO_2 redution. The CO production rates of BNT-P, BNT-OV2 and BNT-OVP are 3.23，5.29 and 3.73 μmol g^{-1} h^{-1}, respectively, under simulated solar light.

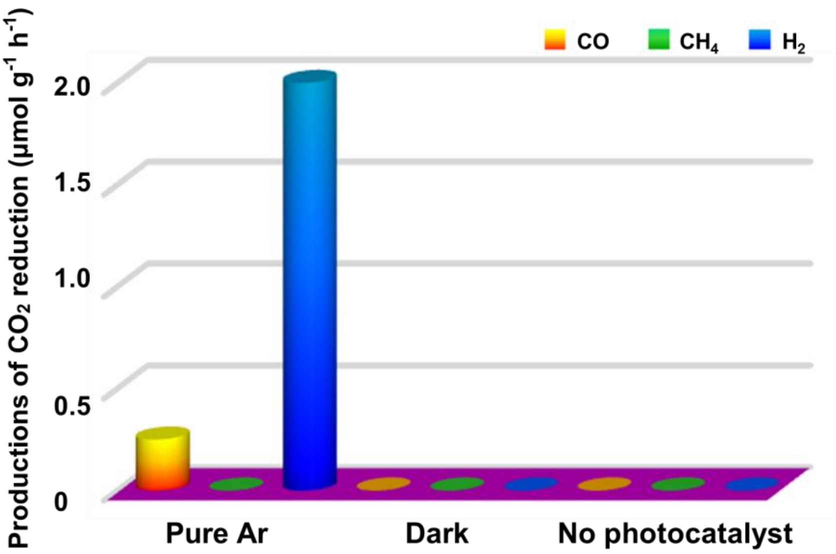

Supplementary Figure 13. Photocatalytic CO_2 reduction rates of BNT-OVP with highly-

pure Ar instead of CO_2, in the darkness and without photocatalyst.

In order to exclude the possibility of influence of organic impurities on the surface of samples, Ar is purged into the reactor instead of CO_2 during the photoreaction, no CH_4 and few CO can be detected in the photocatalytic CO_2 reduction process. Impressively, a great increase in the H_2 production without CO_2 shows that CO_2 molecules are easier to participate in the reaction than water on Bi_3TiNbO_9 nanosheets. No CO can be detected in the dark or without photocatalysts, further confirming the vital role of BNT-OVP catalyst.

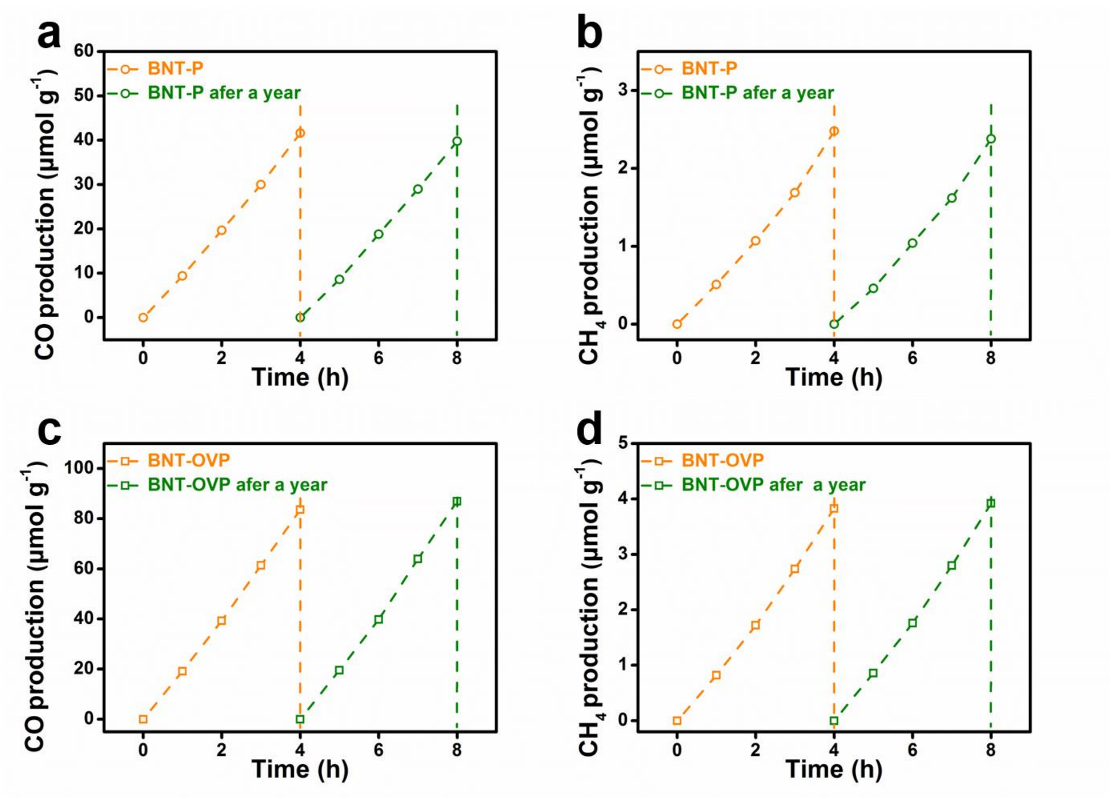

Supplementary Figure 14. (a-d) CO and CH_4 production curves over BNT-OVP and BNT-P after a year.

Notably, for BNT-P and BNT-OVP, the excellent durability of the polarization-induced electric field is revealed by the stable photocatalytic CO_2 reduction performance even after storage of one year.

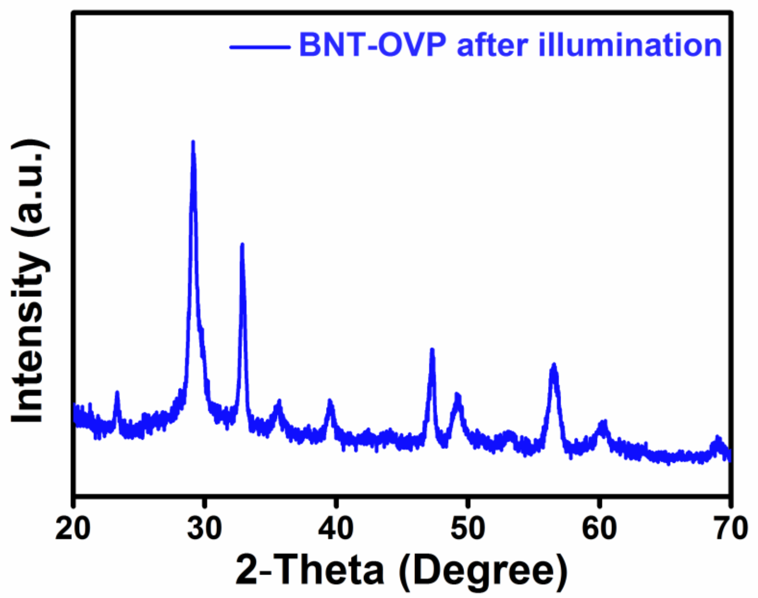

Supplementary Figure 15. XRD patterns of BNT-OVP after photoreaction.

The unchanged XRD pattern after photoreaction reflects the high structural stability of BNT-OVP.

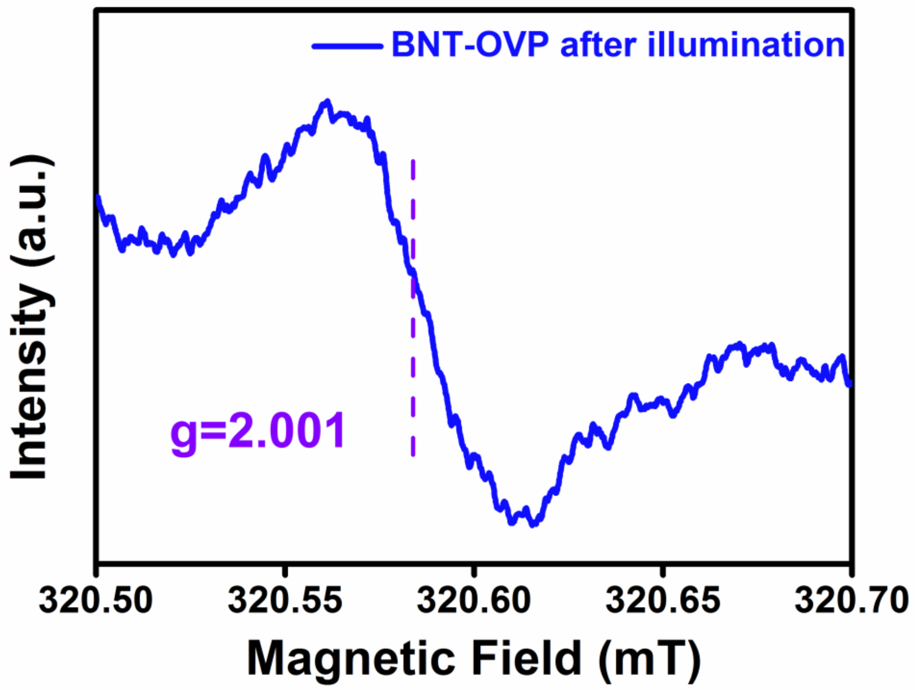

Supplementary Figure 16. EPR spectra of BNT-OVP after photoreaction.

The unchanged EPR spectra after photoreaction reflect the high stability of OVs on BNT-OVP. photoreaction

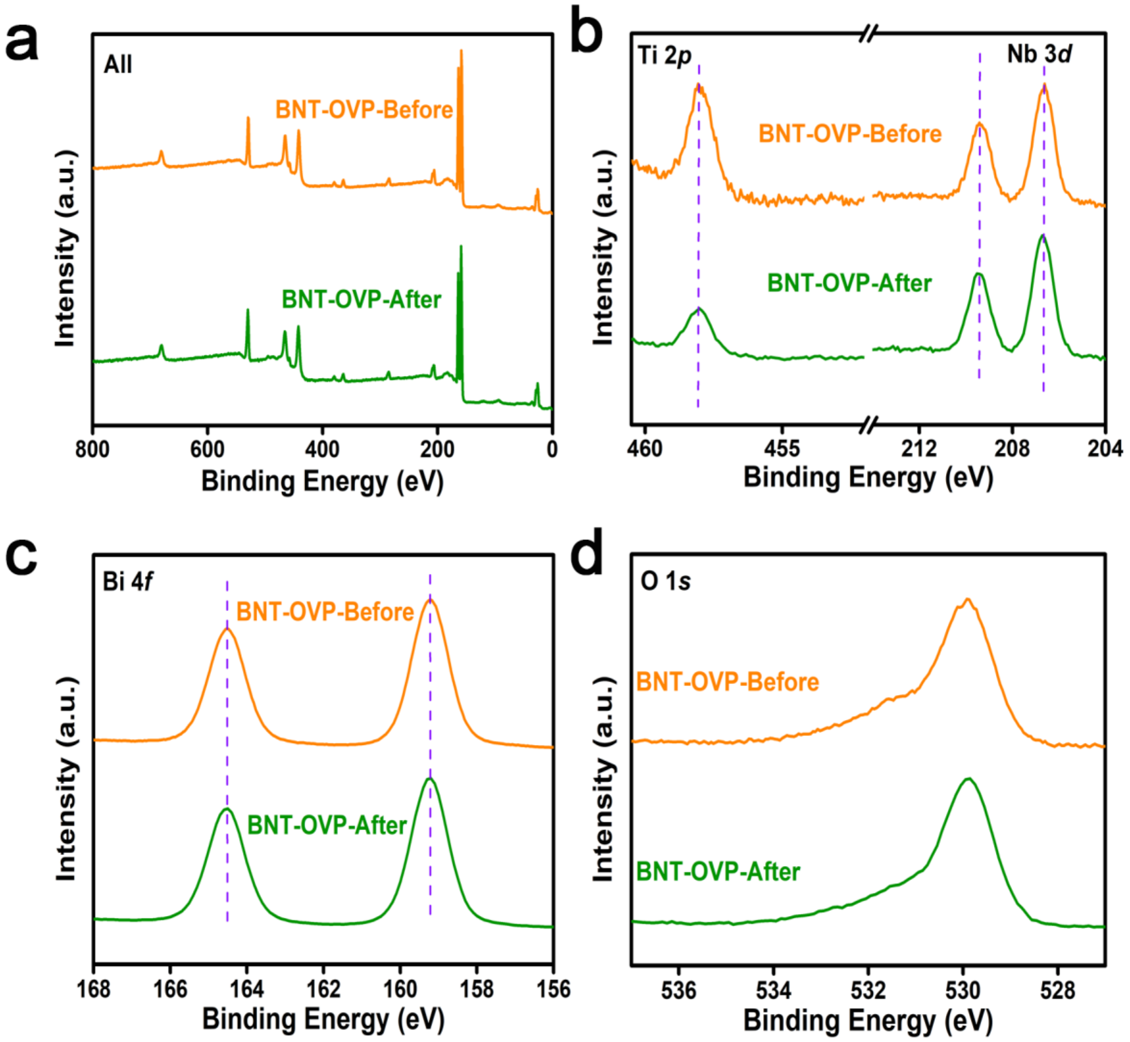

Supplementary Figure 17. (a) Survey XPS spectra, (b) Nd 3*d* and Ti 2*p*, (c) Bi 4*f*, and (d) O1*s* XPS spectra of BNT-OVP and BNT-OVP after photocatalytic CO_2 reduction.

The unchanged XPS spectra after photoreaction reflect the high stability of surface state of BNT-OVP.

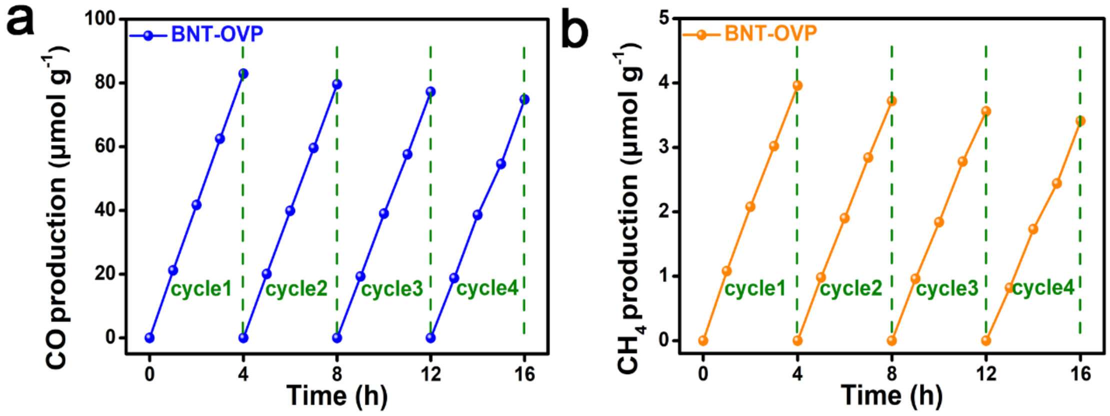

Supplementary Figure 18. The cycling tests of photocatalytic CO_2 reduction into CO (a) and CH_4 (b) over BNT-OVP.

BNT-OVP displays high durability for CO and CH_4 production with negligible activity decay after four consecutive cycles.

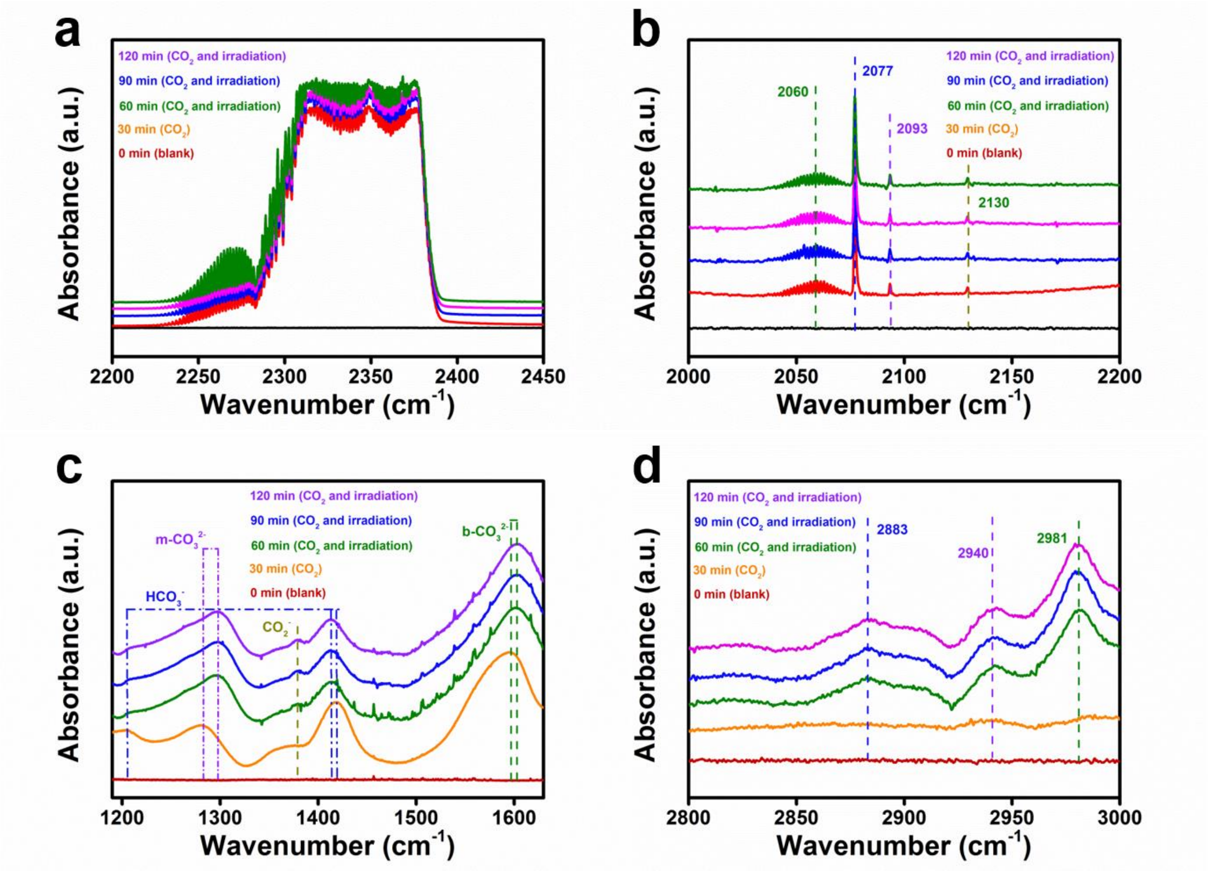

Supplementary Figure 19. (a-d) *In situ* FTIR spectra of BNT-OVP during CO_2 adsorption (0–30 min) and photoreduction (30–120min) processes in the range of 1200-3000 cm^{-1}.

To further analyze CO_2 interaction with BNT-OVP *in-situ* FT-IR experiments were carried out. The strong peaks at around 2300 cm^{-1} belong to the asymmetric stretching of CO_2 underlining the favourable adsorption of CO_2 on BNT-OVP (Supplementary Figure 19a). While the peaks (2060, 2077, 2093 and 2130 cm^{-1}) resulted by the characteristic absorption of CO molecules on BNT-OVP, demonstrating that CO_2 is converted into CO (Supplementary Figure 19b).[3] Thus, CO_2 is constantly transformed into active species, such as carboxylate (CO_2^-, 1298 cm^{-1}), bidentate carbonate (b-CO_3^{2-}, 1381 and 1602 cm^{-1}), bicarbonate (HCO_3^-, 1205 and 1418 cm^{-1}), *HCOO (2883 cm^{-1}) and bidentate formate (2940 and 2981 cm^{-1}) eventually converted into CO and CH_4 during illumination (Supplementary Figure 19c and d).[4, 5] Though the characteristic peaks of active species are slightly shifted, similar carbon active species are generated on both the CO_2 adsorption (initial 0–30 min) the photoreduction (following 30–120 min) processes.

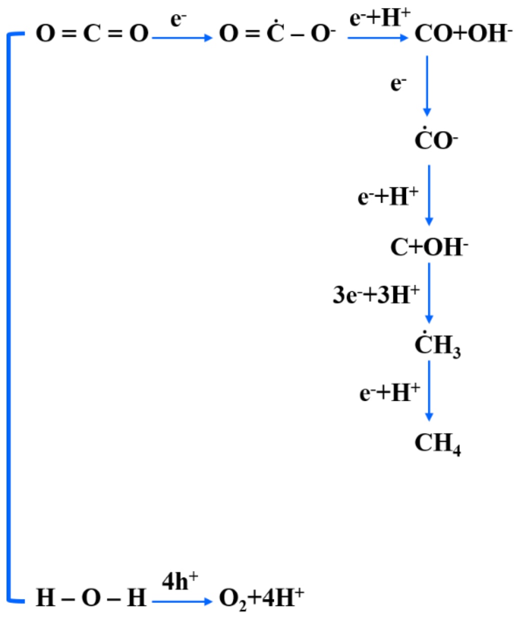

Supplementary Figure 20. Schematic illustration for electron/proton transport process and the formation of product.

The possible electron/proton transport processes and their formation processes are speculated to discuss the reaction mechanism of CO_2 reduction to CO and CH_4. CO_2 molecules are easily adsorbed on the surface of Bi_3TiNbO_9 nanosheets, and then further accept electrons and H^+ to convert into CO. The corona poling process enhances the ferroelectric properties of the samples, and the band bending makes the photo-generated electrons obtain higher reducing ability. Some CO molecules can accept two additional electrons, leading to carbon residue on the surface. These radicals can subsequently combine with up to four electrons and H^+

eventually forming CH_4.

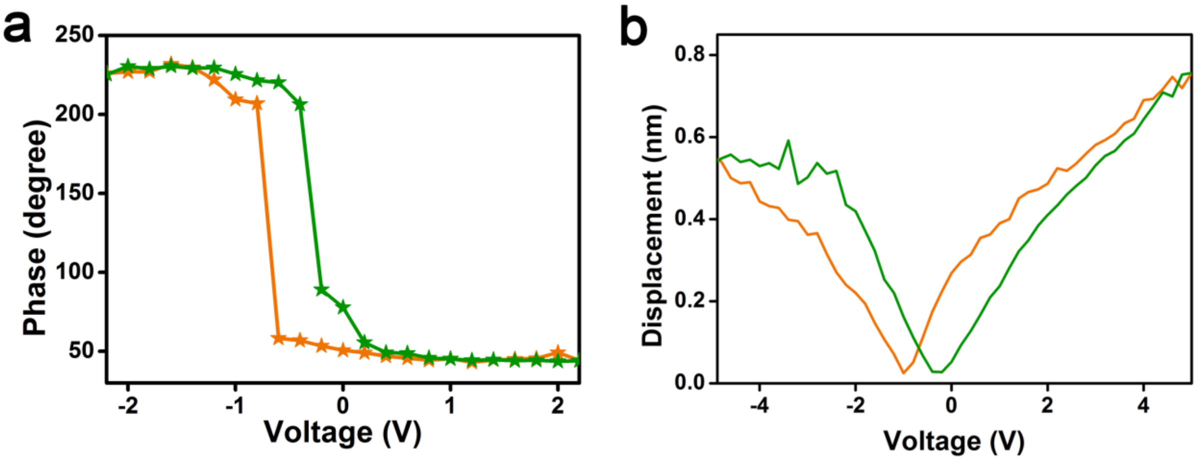

Supplementary Figure 21. (a) Ferroelectric amplitude curve of BNT. (b) The butterfly curves of the piezoelectric response of BNT.

Local polarization-induced electric field loops (P-E) of Bi_3TiNbO_9 nanosheets present a clear hysteresis and 180° phase switch by reversing the external electric field (Supplementary Figure 21a), which indicates a strong response of ferroelectric features. The butterfly curves also confirm the excellent piezoelectric properties of BNT (Supplementary Figure 21b).

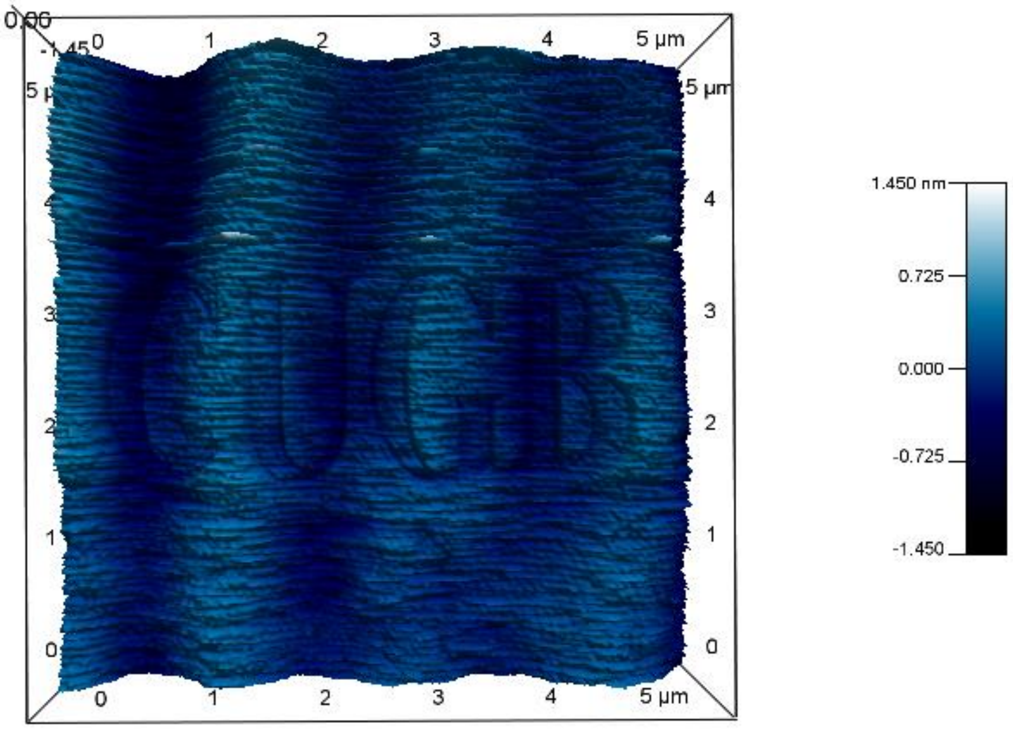

Supplementary Figure 22. AFM 3D image measured in air after applying +10 V and −10 V

voltage of BNT.

Atomic force microscope (AFM) images show a clear CUGB pattern contributed to the domain switching after applying +10 V and −10 V voltage in different zones of Bi_3TiNbO_9 nanosheets.

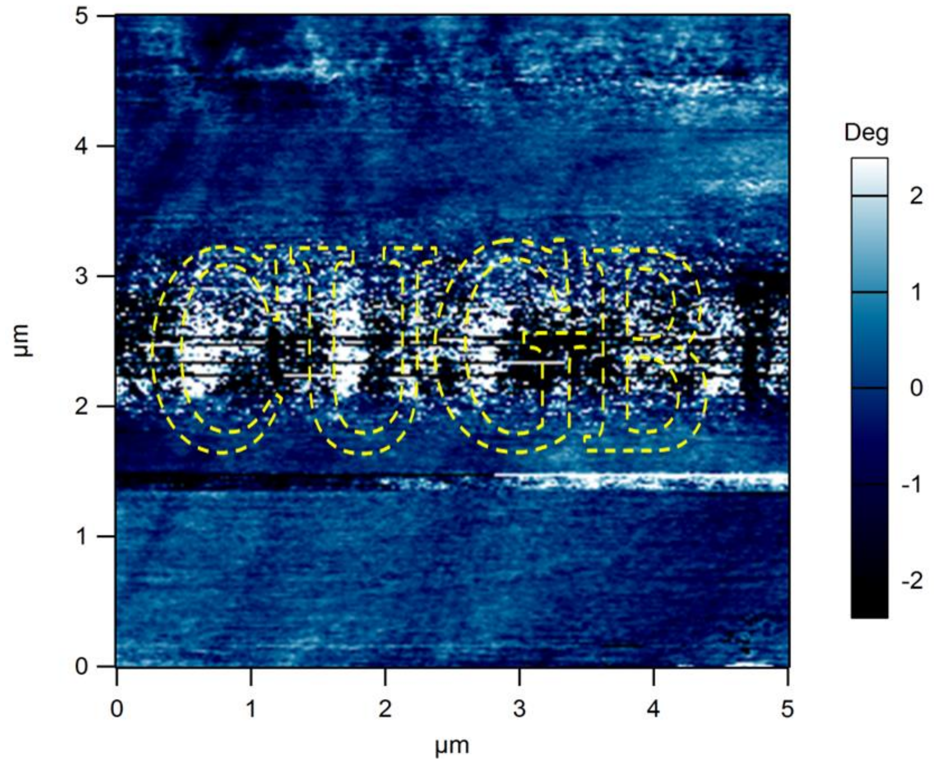

Supplementary Figure 23. The standard ferroelectric phase image of BNT measured in air after applying +10 V and −10 V voltage.

Piezoresponse force microscopy (PFM) exhibits two levels of dark and bright regions, illustrating the negatively and positively polarized domains by different voltages, respectively. Due to limited voltage, the unclear phase suggests unstable ferroelectric switching under low voltage.

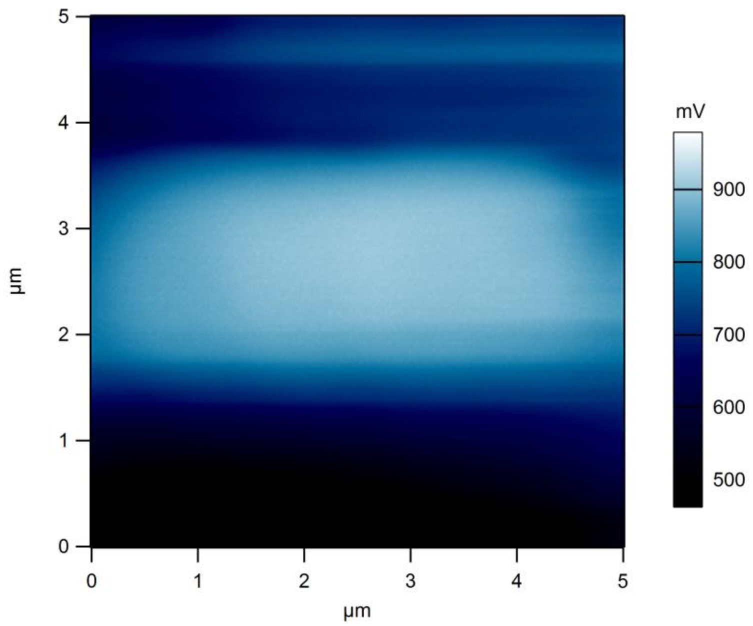

Supplementary Figure 24. Surface charge of BNT measured in air after applying +10 V and −10 V voltage.

The obtained surface charge images show a heterogeneous charge distribution on the surface of BNT, in line with a polarization-induced electric field formed between dark and bright regions.

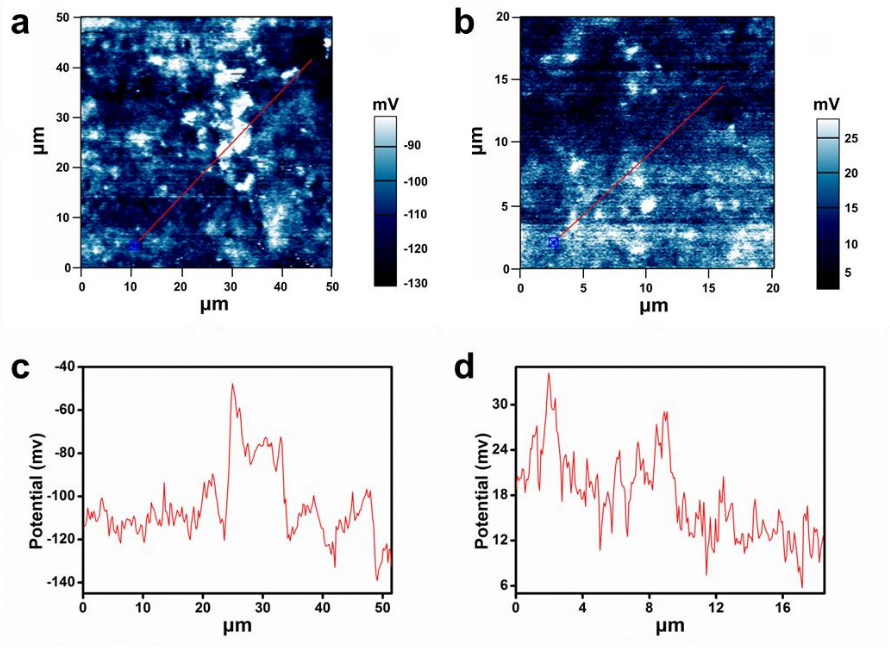

Supplementary Figure 25. Surface charge and corresponding charge difference profile of (a, c) BNT-OVP and (b, d) BNT-P.

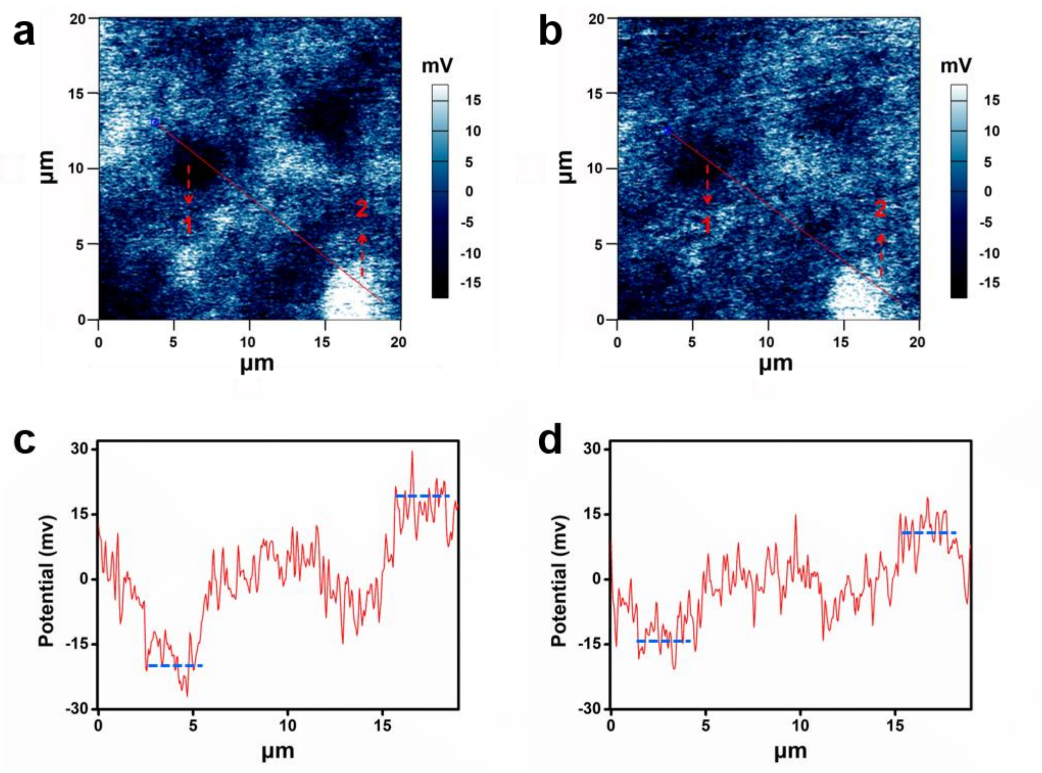

Supplementary Figure 26. Surface charge and corresponding charge difference profile of BNT-OVP (a, c) in the dark and (b, d) under irradiation.

The surface charge potential of BNT-OVP can be ascribed to the positive polarization and negative polarization with dark and bright regions due to the polarization-induced electric field. It is remarkable that BNT-OVP shows a ~5 mV decrease at the region of positive and negative potentials under the condition of illumination, which is caused by that the photogenerated electrons and holes are separated and transferred to the two opposite direction.

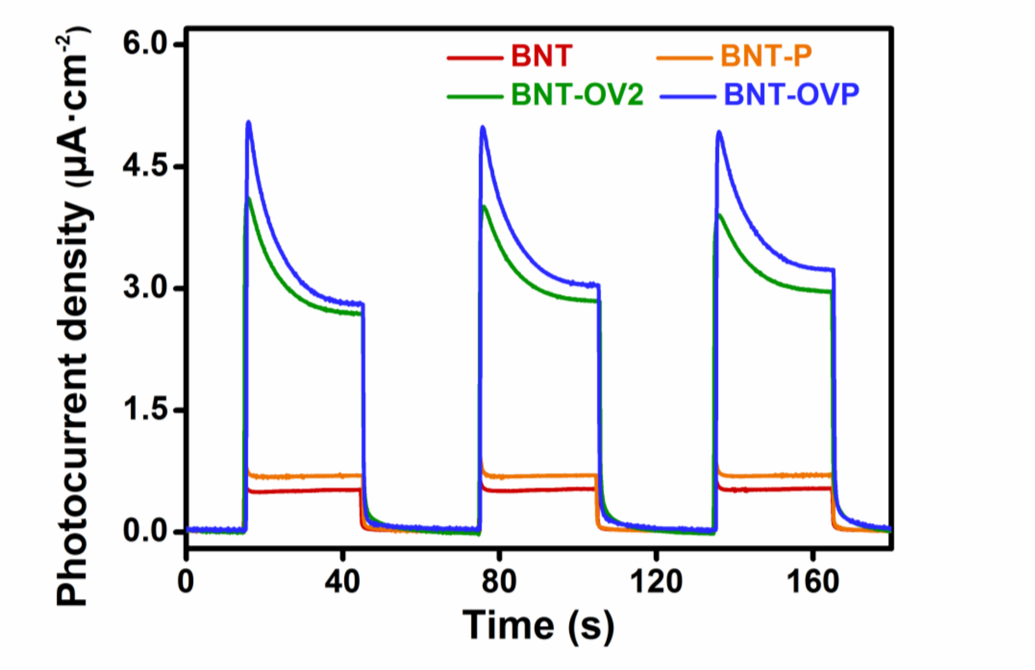

Supplementary Figure 27. Photocurrent density of BNT, BNT-P, BNT-OV2 and BNT-OVP under simulated solar light.

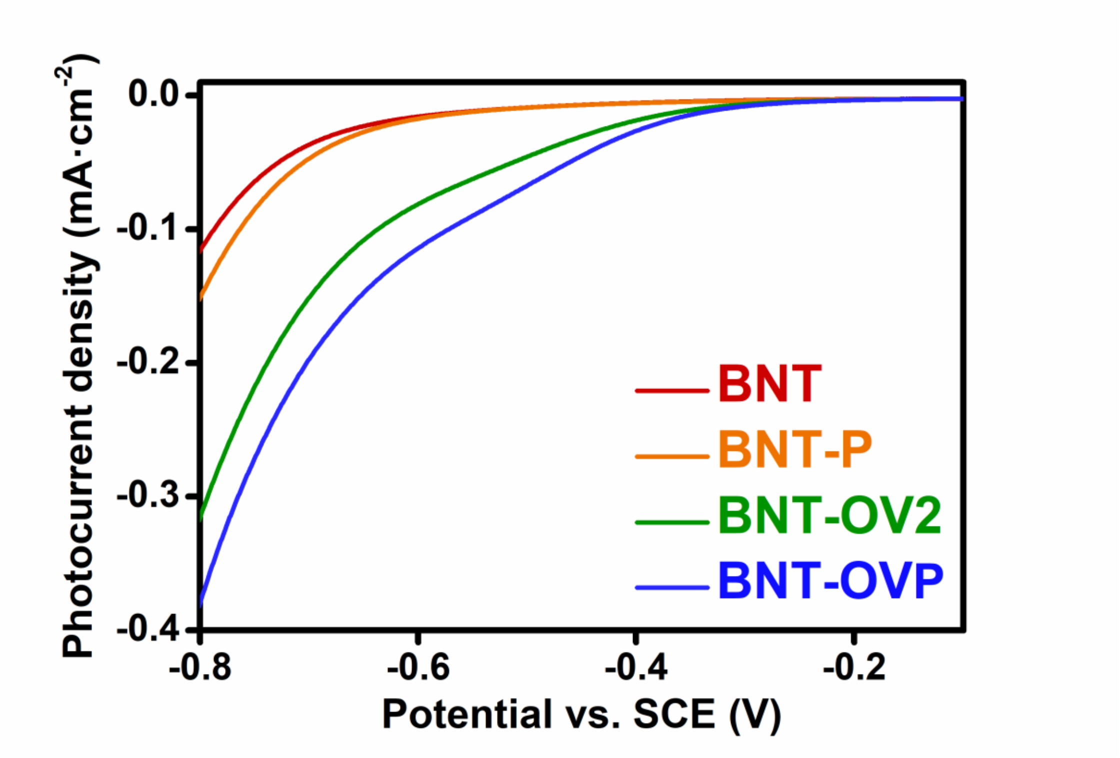

Supplementary Figure 28. I–V curves of BNT, BNT-P, BNT-OV2 and BNT-OVP.

BNT-OVP displays the strongest photocurrent, confirming its highest charge separation efficiency.

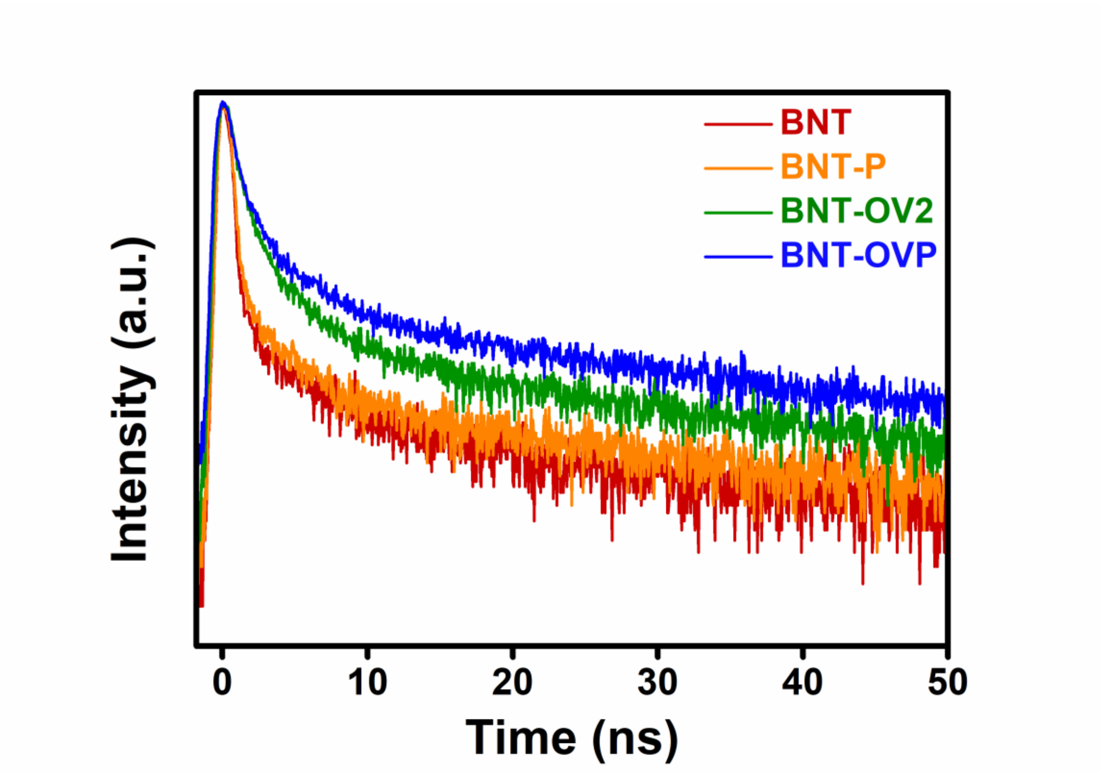

Supplementary Figure 29. Time resolved PL curves for BNT, BNT-P, BNT-OV2 and BNT-OVP.

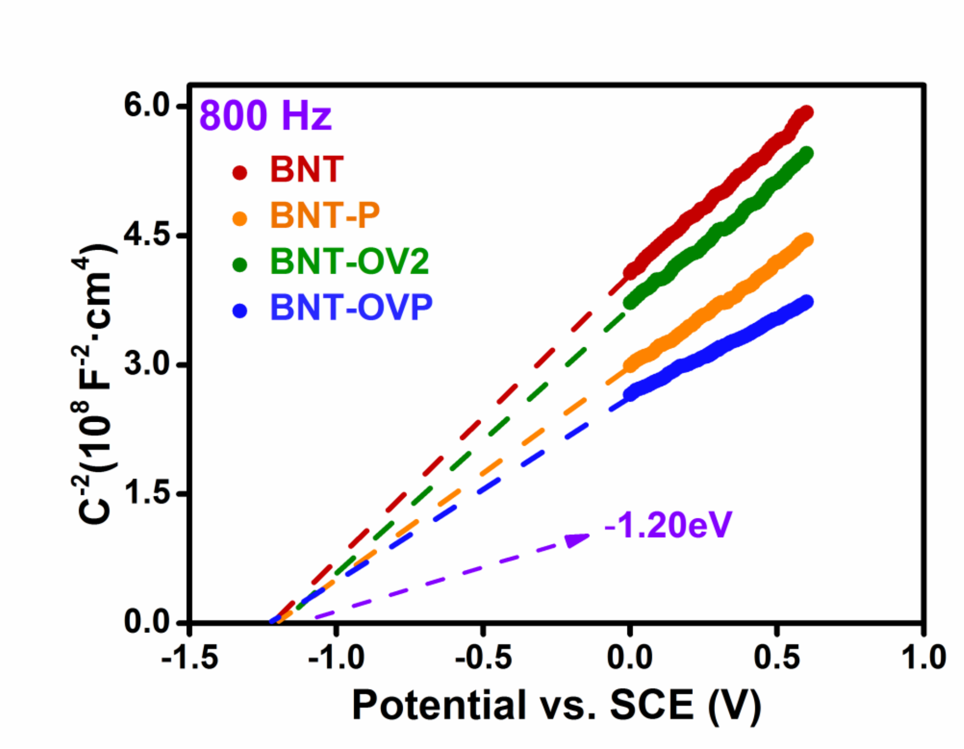

Supplementary Figure 30. Mott–Schottky plots of BNT, BNT-P, BNT-OV2 and BNT-OVP at a frequency of 800 Hz (0.1 M Na_2SO_4).

Mott–Schottky plots at a frequency of 800 Hz show that BNT-OVP dispalys the highest carrier density compared with BNT, BNT-P and BNT-OV2.

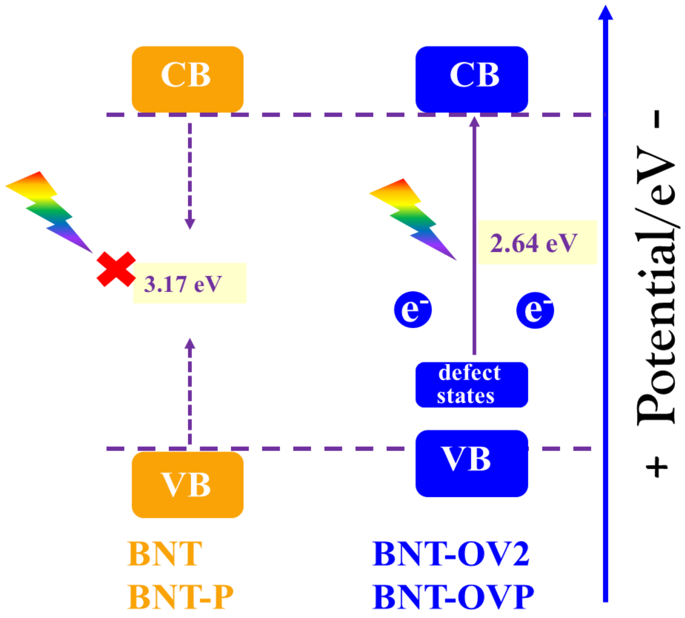

Supplementary Figure 31. Schematic band structure of BNT, BNT-P, BNT-OV2 and BNT-OVP.

Based on the above results, a schematic diagram is used to describe the band structure of BNT, BNT-P, BNT-OV2 and BNT-OVP. OV introduces defect states between CB and VB, benefitting the transition of electrons. What's more, compared with BNT and BNT-P, BNT-OV2 and BNT-OVP have a narrower band gap to absorb more visible light.

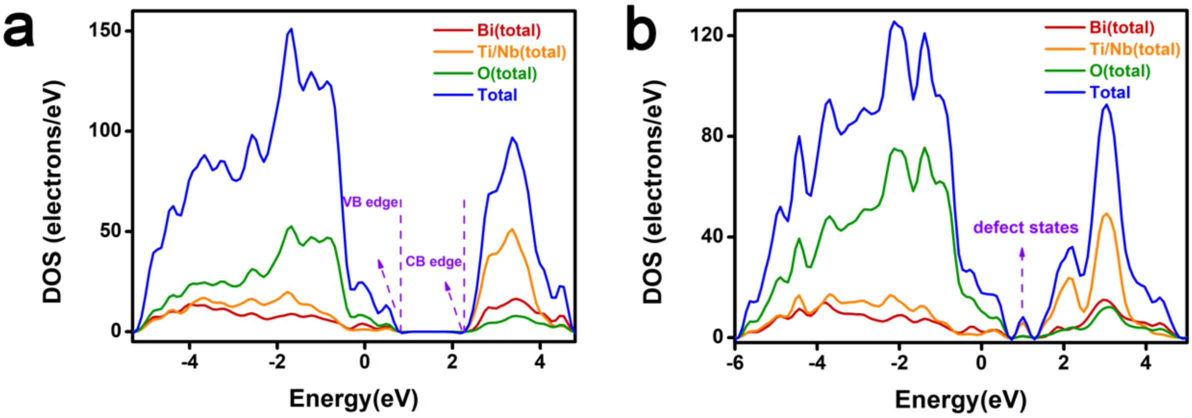

Supplementary Figure 32. (a, b) Densities of states (DOS) of Bi_3TiNbO_9 and Bi_3TiNbO_9 with OV

Density of states (DOS) of Bi_3TiNbO_9 is calculated by density functional theory (DFT), which show that the valence band maximum (VBM) of Bi_3TiNbO_9 mainly consists of O-2p orbital, while the conduction band minimum (CBM) is composed of Ti-3d and Nb-3d orbitals. Significantly, the introduced oxygen vacancy leads to a new defect level in the band gap, which is beneficial to photoexcitation and charge separation.

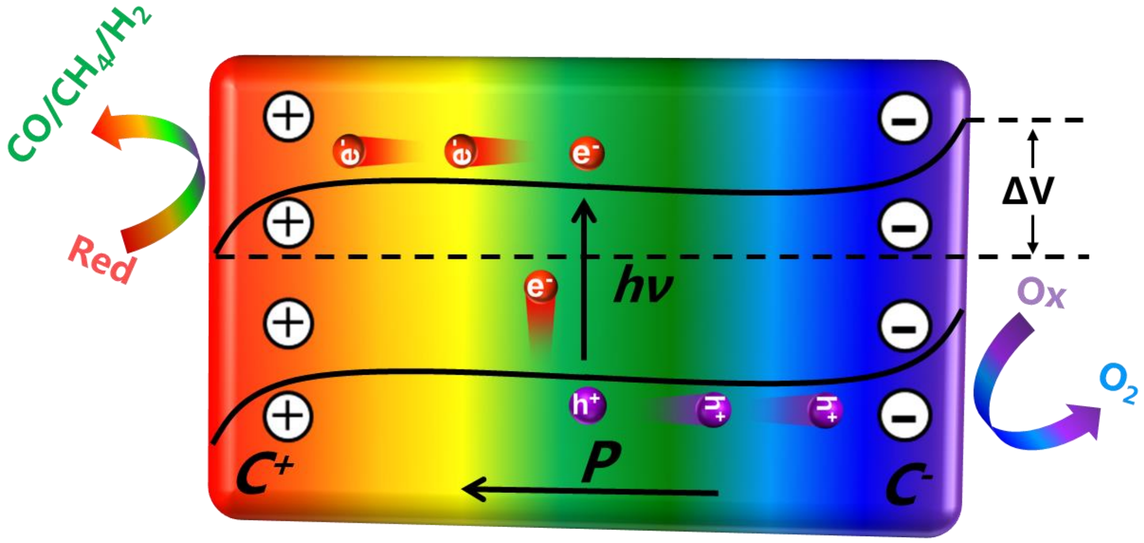

Supplementary Figure 33. Schematic illustration of the ferroelectric catalysis mechanism and tilting of energy bands under polarization field and the accompanied redox reactions.

The domains gradually switch to be aligned after corona poling, resulting in a larger potential difference caused by band bending, which provides a stronger driving force for charge separation and higher photocatalytic activity.

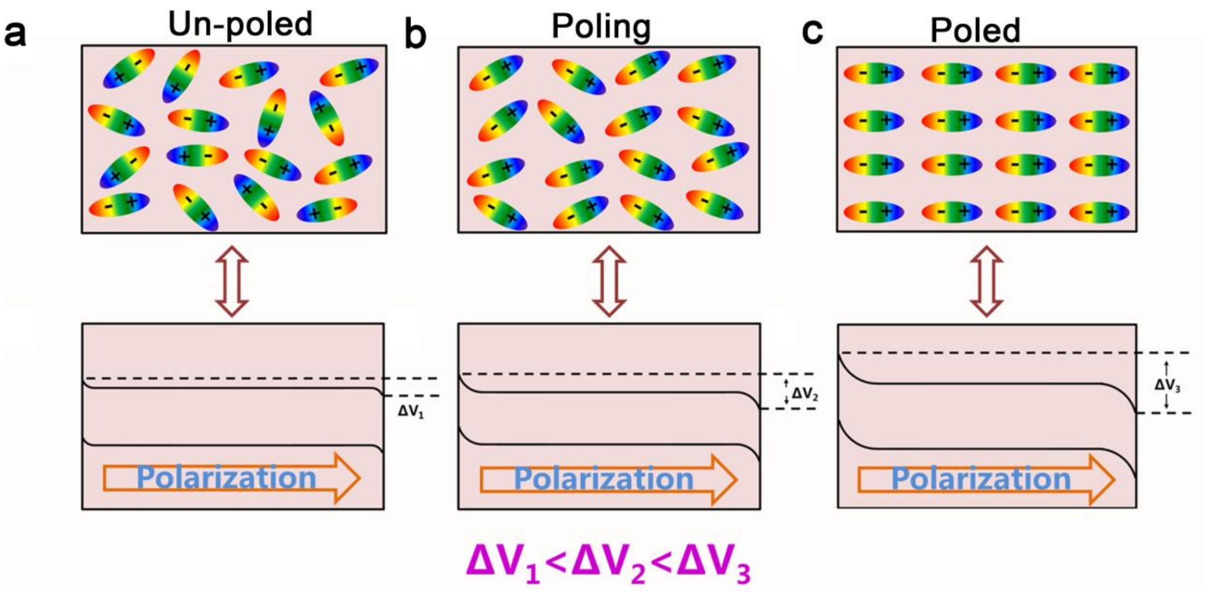

Supplementary Figure 34. Scheme for dipole moment of Bi_3NbTiO_9 sheets: (a) un-poled, (b) poling, (c) fully poled and corresponding tilting of energy bands.

The positively polarized charges can bend down the energy band to promote the reduction reaction, while the negatively polarized charges can bend up the energy band to enhance the oxidation reaction, which effectively improve the photocatalytic CO_2 reduction activity of Bi_3NbTiO_9 nanosheets. With the increase of poling voltage, the domains gradually switch to be aligned, resulting in a larger potential difference caused by band bending, which provides a stronger driving force for charge separation and higher photocatalytic activity.

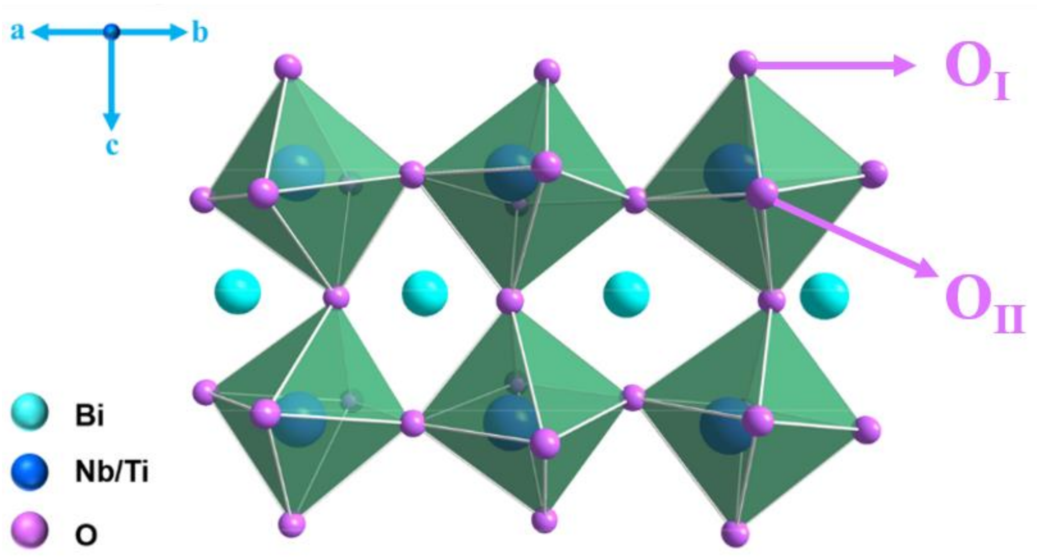

Supplementary Figure 35. Unit cell structure of Nb/Ti-centered octahedra perovskite slabs in Bi_3NbTiO_9.

There are main two nonequivalent oxygen sites in the Nb/Ti-centered octahedra in Bi_3NbTiO_9, namely, that in the direction perpendicular and parallel to the ferroelectric polarization, which is labled as O_I and O_{II}, respectively.

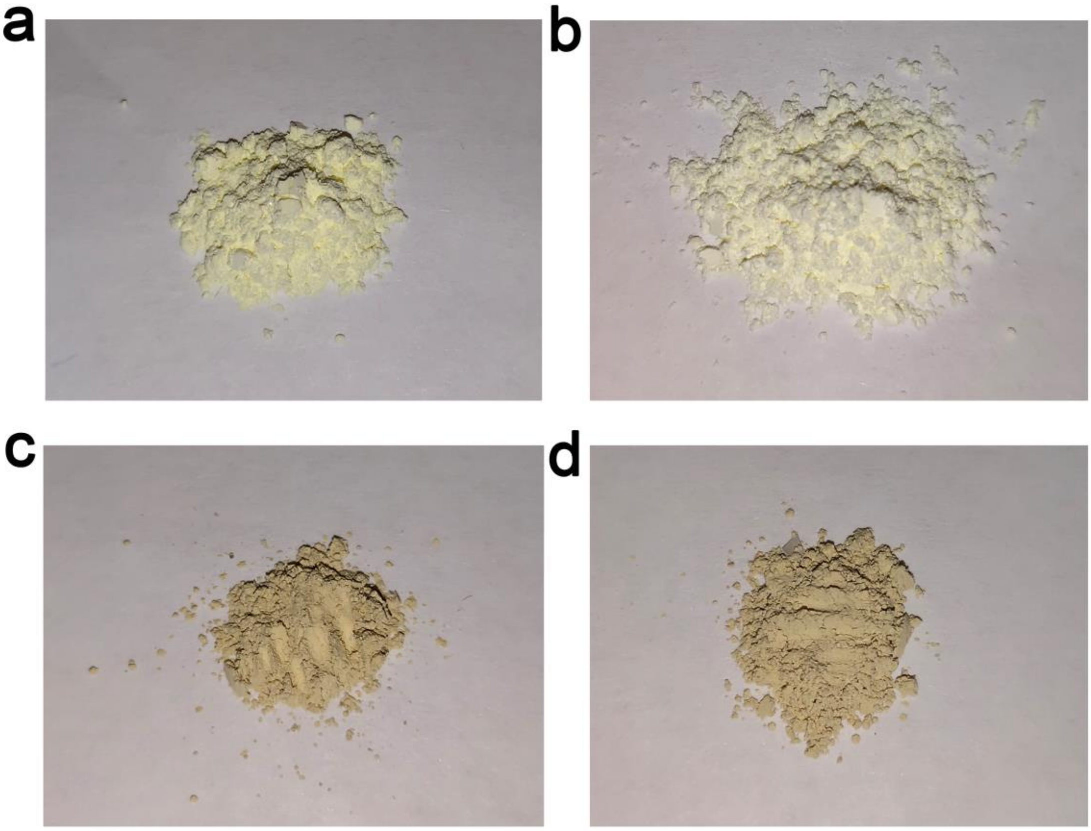

Supplementary Figure 36. Pictures of (a) BNT, (b) BNT-P, (c) BNT-OV2 and (d) BNT-OVP samples.

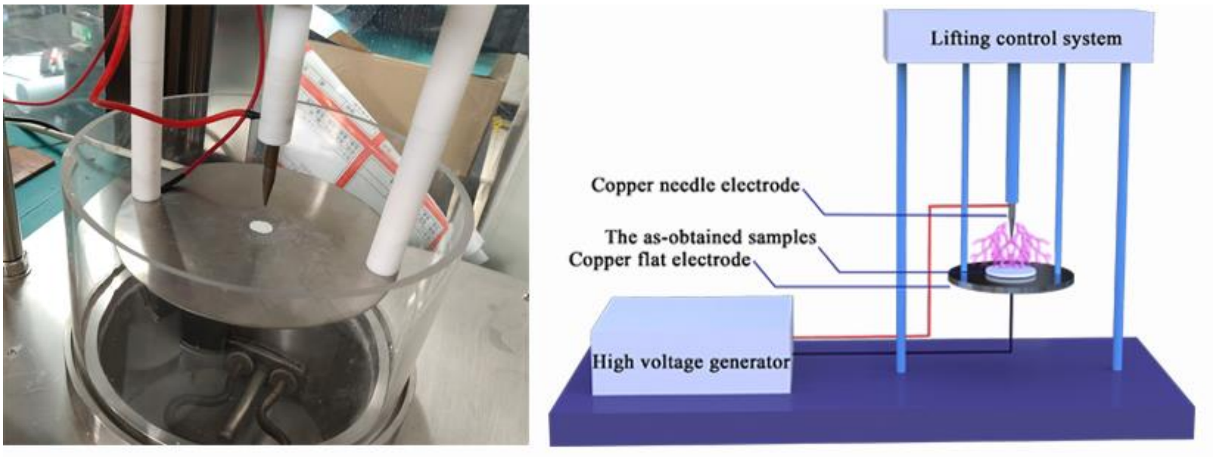

Supplementary Figure 37. The pictures of corona poling device and the schematic illustration for corona poling process.

50 mg of BNT or BNT-OV2 powder is uniformly coated on a negative disk-like copper electrode with an area of about 3 cm2. And the voltage of the steel-point electrode is 20 kV with a 1cm distance between the two electrodes for 30 min to obtain polarized BNT-P or BNT-OVP, respectively.

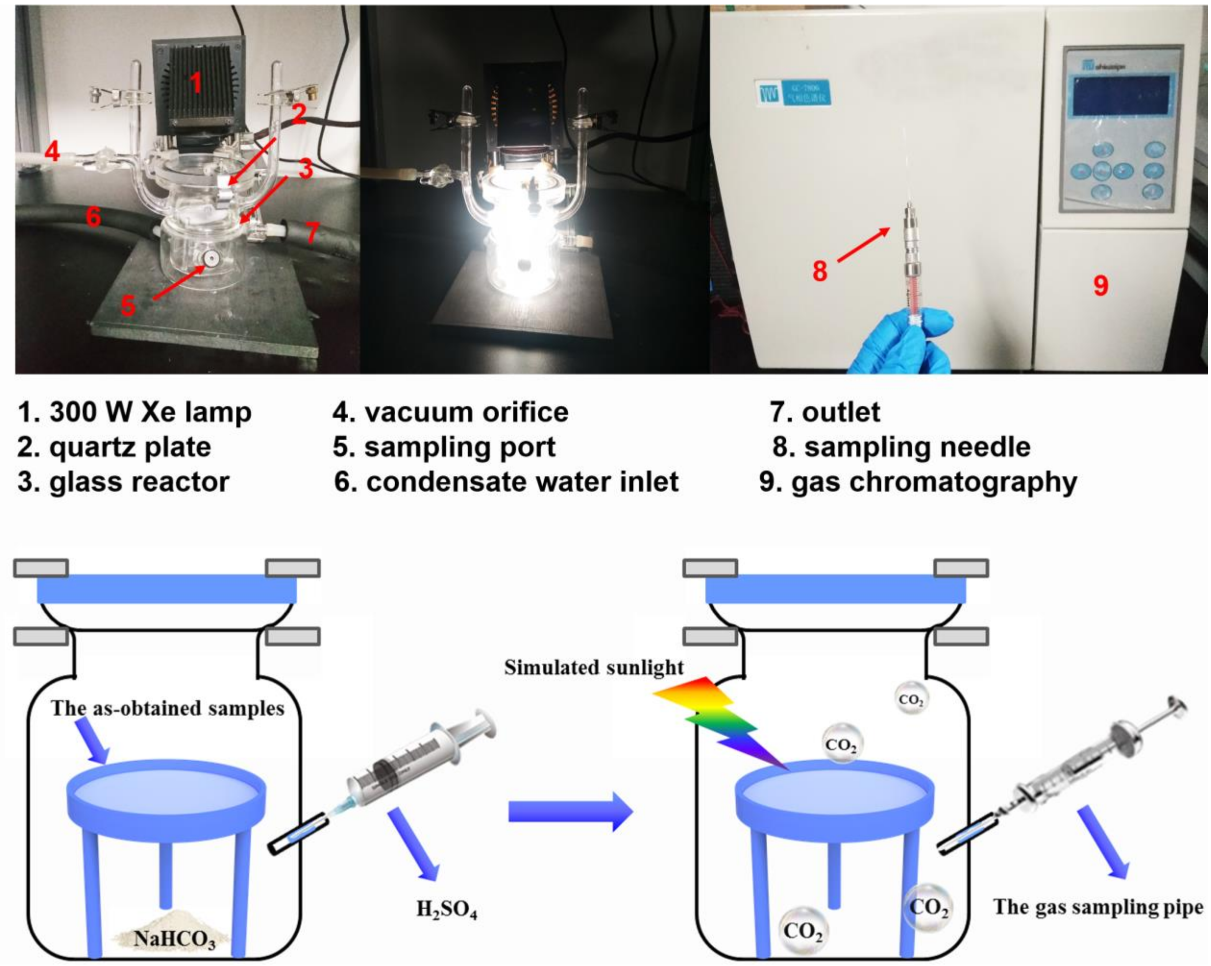

Supplementary Figure 38. The pictures of the photocatalytic CO_2 reduction system used in this work and the corresponding scheme.

Supplementary Table 1. Comparison of the activity of BNT-OVP in the photocatalytic CO_2 reduction with the catalysts reported in literatures.[6]

Photocatalyst	Catalyst amount (mg); Reaction solution (mL)	Light source	Products	Evolution rate (μmol g^{-1} h^{-1})	Reference
BNT-OVP	50mg<br>5ml (4M H_2SO_4)	300 W Xe lamp	CH_4<br>CO	CH_4: 0.96<br>CO: 20.91	This work
BiOCl	100mg<br>100ml (deionized water)	500 W Xe lamp	CH_4<br>CO	CH_4: 0.15<br>CO: 1.01	7
BiOBr	50mg<br>5ml (4M H_2SO_4)	Xe lamp with light intensity of 0.21 W/cm^2	CH_4<br>CO	CH_4: ~0<br>CO: 4.45	8
BiOI	50mg<br>5ml (4M H_2SO_4)	300 W Xe lamp	CH_4<br>CO	CH_4: 1.78<br>CO: 5.18	9
$Bi_4O_5Br_2$	150mg<br>5ml (4M H_2SO_4)	300 W Xe lamp<br>(λ> 400 nm)	CH_4<br>CO	CH_4: 2.04<br>CO:2.74	10
$Sr_2Bi_2Nb_2TiO_{12}$	50mg<br>5ml (4M H_2SO_4)	300 W Xe lamp	CO	CO:17.11	6
$BiOIO_3$	20mg<br>5ml (4M H_2SO_4)	300 W Xe lamp	CH_4<br>CO	CH_4： ~0.02<br>CO: 17.33	11
Br-$Bi_2O_2(OH)(NO_3)$	20mg<br>15ml (1.33M H_2SO_4)	300 W Xe lamp	CO	CO:8.12	12
Bi_5O_7I	50mg<br>5ml (4M H_2SO_4)	300 W Xe lamp<br>(λ> 400 nm)	CH_4<br>CO	CH_4: 0.18<br>CO:1.73	13
$Bi_4O_5I_2$	50mg<br>5ml (4M H_2SO_4)	300 W Xe lamp<br>(λ> 400 nm)	CH_4<br>CO	CH_4: 0.22<br>CO:19.82	14
Bi_2WO_6	100mg<br>1ml (deionized water)	300 W Xe Lamp (λ> 420 nm)	CH_4<br>CO	CH_4: 1.1<br>CO: 0	15
Pt/Bi_2MoO_6	100mg<br>0.4ml (deionized water)	300 W Xe lamp	CH_4<br>CO	CH_4: 0.29<br>CO: 0	13
$(BiO)_2CO_3$	100 mg;<br>1 mL<br>(pure water)	300 W Xe lamp<br>(λ> 400 nm)	CO	CO:3.98	16
Mg-In LDH	100 mg;<br>4 mL<br>(pure water)	200 W Hg-Xe lamp	CH_4<br>CO	CH_4: 0<br>CO:4	17
$SrNb_2O_6$	10mg<br>4 mL water	Xe lamp (λ=300–780 nm)	CH_4<br>CO	CH_4: 3.3<br>CO:16.6	18
Cu_2S	100 mg(pure water)	Xe lamp(450 W)	CO	CO: 3.02	19
Cu/TiO_2	20 mg<br>5 mL (0.5 M H_2SO_4)	Xe lamp(500 W)	CH_4<br>CO	CH_4:0.13<br>CO: 0.80	20

Supplementary Table 2. Time resolved PL decay parameter for BNT, BNT-P, BNT-OV2 and BNT-OVP.

	τ_1 (ns)	A_1	τ_2 (ns)	A_2	τ_{avg} (ns)
BNT	0.47	16934.6	11.05	78.1	1.51
BNT-P	0.52	10152	11.07	91.4	2.22
BNT-OV2	1.02	4261	10.50	203.5	4.14
BNT-OVP	1.04	2511.1	12.62	246.1	7.33

2. References

1. Zhang, J. Z. et al. Lattice dynamics, dielectric constants, and phase diagram of bismuth layered ferroelectric $Bi_3Ti_{1-x}W_xNbO_{9+\delta}$ ceramics. J. Am. Ceram. Soc. 99, 3610–3615 (2016).

2. Sun, S. J. & Yin, X. F. Engineered layer-stacked interfaces inside Aurivillius-type layered oxides enables superior ferroelectric property. Crystals 10, 710 (2020).

3. Yang, S.W. et al. FTIR study of CO and NO adsorbed on nitrided CoMo/Al_2O_3 catalysts. *Phys. Chem. Chem. Phys*. **2,** 3313–3317 (2000). DOI: 10.1039/b002557g.

4. Ma, Z. Y. et al. Oxygen Vacancies induced exciton dissociation of flexible BiOCl nanosheets for effective photocatalytic CO_2 conversion. *J. Mater. Chen. A* **5,** 24995-25004 (2017). https://doi.org/10.1039/C7TA08766G.

5. Liu, L. J. et al. Engineering Coexposed {001} and {101} Facets in Oxygen-Deficient TiO_2 Nanocrystals for Enhanced CO_2 Photoreduction under Visible Light. *ACS Catal*. **6,** 1091–1108 (2016). https://doi.org/10.1021/acscatal.5b02098.

6. Yu, H. J. et al. Three-in-One Oxygen Vacancies: Whole Visible-Spectrum Absorption, Efficient Charge Separation, and Surface Site Activation for Robust CO_2 Photoreduction. *Angew. Chem. Int. Ed..58*, 3880-3884 (2019). https://doi.org/10.1002/anie.201813967.

7. Zhang, L. et al. Photoreduction of CO_2 on BiOCI nanoplates with the assistance of

photoinduced oxygen vacancies. *Nano Res.* **8**, 821-831 (2015). DOI: 10.1007/s12274-014-0564-2.

8. Wu, D., Ye, L.Q., Yip, H. Y. & Wong, P. K. Organic-free synthesis of {001} facets dominated BiOBr nanosheets for selective photoreduction of CO_2 to CO. *Catal. Sci. Technol.* **7**, 265-271 (2016). DOI: 10.1039/c6cy02040b.

9. Ye, L. Q. et al. Facet-dependent photocatalytic reduction of CO_2 on BiOI nanosheets. *Chem. Eng. J.* **291**, 39-46 (2016). DOI: 10.1016/j.cej.2016.01.032.

10. Ye, L. Q. et al. Thickness-ultrathin and bismuth-rich strategies for BiOBr to enhance photoreduction of CO_2 into solar fuels. *Appl. Catal. B: Environ.* **187**, 281-290 (2016). DOI: 10.1016/j.apcatb.2016.01.044.

11. Chen, F. et al. Macroscopic spontaneous polarization and surface oxygen vacancies collaboratively boosting CO_2 photoreduction on $BiOIO_3$ single crystals. Adv. Mater. (2020).

12. Hao, L. et al. Surface halogenation induced atomic site activation and local charge separation for superb CO_2 photoreduction. *Adv. Mater.* **31,** 1900546 (2019). DOI: 10.1002/ adma.201900546.

13. Ding, C. H. et al. Synthesis of $Bi_xO_yI_z$ from molecular precursor and selective photoreduction of CO_2 into CO. *J. CO_2 Util.* **14**, 135-142 (2016). DOI: 10.1016/j.jcou.2016.04.012.

14. Zhou, Y. et al. High-yield synthesis of ultrathin and uniform Bi_2WO_6 square nanoplates benefitting from photocatalytic reduction of CO_2 into renewable hydrocarbon fuel under visible light. *Acs. Appl. Mater. Inter.* **3**, 3594-3601 (2011). DOI: 10.1021/am2008147.

15. Zhang, Y. et al. Bi_2MoO_6 nanostrip networks for enhanced visible-light photocatalytic reduction of CO_2 to CH_4. *ChemPhysChem*. **18**, 1-6 (2017). DOI: 10.1002/cphc.201700655.

16. Yang, H. et al. Synthesis of herarchical $(BiO)_2CO_3$ nanosheets microspheres toward efficient photocatalystic reduction of CO_2 into CO. *Phys. E.* **78**, 100-104 (2016). DOI:

10.1016/j.physe.2015.12.001

17. Teramura, K. et al. Photocatalytic conversion of CO. *Angew. Chem. Int. Ed.* **51**, 8008-8011 (2012). DOI: 10.1002/ange.201201847.

18. Xie, S. J. et al. $SrNb_2O_6$ nanoplates as efficient photocatalysts for the preferential reduction of CO_2 in the presence of H_2O. *Chem. Commun.* **51**, 3430-3433 (2015). DOI: 10.1039/c4cc10241j.

19. Manzi, A. et al. Light-Induced Cation Exchange for Copper Sulfide Based CO_2 Reduction. *J. Am. Chem. Soc.*, **137,** 14007-14010 (2015).

20. Zhao, J., Li, Y.X., Zhu, Y.Q., Wang, Y. & Wang, C.Y. Enhanced CO_2 photoreduction activity of black TiO_2-coated Cu nanoparticles under visible light irradiation: Role of metallic Cu. *Appl. Catal. A-Gen,* **510,** 34-41 (2016). DOI: 10.1016/j.apcata.2015.11.001.
